# Sind die Ergebnisse von Knietotalendoprothesen nach Tibiakopfumstellungsosteotomie schlechter?

**DOI:** 10.1007/s00132-021-04134-4

**Published:** 2021-07-16

**Authors:** Marcel Mäder, Franziska Beyer, Cornelia Lützner, Jörg Lützner

**Affiliations:** UniversitätsCentrum für Orthopädie & Unfallchirurgie, Universitätsklinikum Carl Gustav Carus, TU Dresden, Fetscherstr. 74, 01307 Dresden, Deutschland

**Keywords:** Kniegelenkersatz, totaler, Outcomestudien, Patientenberichtetes Outcome, Patientenzufriedenheit, Bewegungsumfang, Knee replacemant, total, Outcomes studies, Patient reported outcomes, Patient satisfaction, Range of motion

## Abstract

**Hintergrund:**

Bei einem Teil der Patienten nach Tibiakopfumstellungsosteotomie (HTO) wird die Implantation einer Knietotalendoprothese (Knie-TEP) notwendig. Durch die HTO kann die Anatomie ungünstig verändert und die Knie-TEP-Operation erschwert sein. Ziel dieser Studie war es zu untersuchen, ob Patienten nach HTO gegenüber denjenigen mit primärer Gonarthrose in gleichem Maße von einer Knie-TEP profitieren.

**Material und Methoden:**

Im lokalen Register konnten insgesamt 44 Patienten nach HTO und 1703 Patienten mit primärer Gonarthrose identifiziert werden. Zur Reduktion von Confoundern erfolgte eine 1:1 „propensity score matched-pair“-Analyse (Alter, Geschlecht, BMI, Komorbiditäten) bei Patienten mit einem 5‑Jahres-Follow-up. Es resultierten 35 gematchte Paare, welche hinsichtlich Kniefunktion, Schmerzniveau, Zufriedenheit mit dem Operationsergebnis sowie perioperativen Daten (Schnitt-Naht-Zeit, Implantattyp, Komplikationen) und Revisionen verglichen wurden.

**Ergebnisse:**

Patienten mit vorangegangener HTO hatten prä- und 5 Jahre postoperativ eine vergleichbare Kniefunktion, jedoch ein signifikant höheres prä- und postoperatives Schmerzniveau. Trotz des höheren Schmerzniveaus zeigte sich eine vergleichbare Zufriedenheit mit dem Operationsergebnis. Die Schnitt-Naht-Zeit für die Knie-TEP nach HTO war signifikant länger und es wurden signifikant häufiger modulare Endoprothesen mit Stielverankerung implantiert. Hinsichtlich postoperativer Komplikationen innerhalb der ersten 3 Monate nach Operation unterschieden sich beide Kohorten nicht signifikant. Die Revisionsrate innerhalb von 5 Jahren war bei Patienten nach HTO nicht erhöht.

**Schlussfolgerung:**

Fünf Jahre nach der Knie-TEP zeigten Patienten mit vorangegangener HTO eine vergleichbare Kniefunktion wie Patienten mit primärer Gonarthrose. Jedoch war bei Patienten nach HTO ein höheres Schmerzniveau zu verzeichnen. Der Operationsaufwand der Knie-TEP nach HTO war deutlich höher.

## Abstract (en)

**Background:**

In some cases, total knee arthroplasty (TKA) following high tibial osteotomy (HTO) is necessary. HTO can adversely affect anatomy and alignment resulting in more complicated and challenging TKA surgery. The aim of this study was to investigate whether patients having undergone HTO benefit from TKA to the same extent as patients with primary osteoarthritis of the knee.

**Methods:**

A total of 44 patients after HTO and 1703 patients with primary osteoarthritis of the knee were identified in the local registry. To reduce confounders, a 1:1 propensity score matched-pair analysis (age, gender, BMI, comorbidities) was carried out in patients with a 5-year follow-up. This resulted in 35 matched pairs, which were compared for knee function, pain level, satisfaction and perioperative data (operative time, implant type, complications) and revisions.

**Results:**

Patients having undergone HTO had no significant differences in knee function prior and 5 years after surgery but a significantly higher pain level. Despite the higher pain level before and after TKA, there was a similar satisfaction with the result of the surgery. The operative time for TKA was significantly longer after HTO and modular implants with stem and wedges were used significantly more often. The two cohorts did not differ with respect to complications within the first 3 months after surgery. The revision rate within 5 years was not increased in patients after HTO.

**Conclusion:**

Patients having undergone HTO achieved a similar knee function in comparison to patients with primary osteoarthritis of the knee after TKA. However, a higher pre- and postoperative pain level was recorded in patients following HTO. The surgical effort was significantly higher after HTO.

## Einleitung

Die Tibiakopfumstellungsosteotomie (HTO) ist eine gelenkerhaltende chirurgische Maßnahme zur Behandlung biologisch junger Patienten mit hohem funktionellem Anspruch bei medialen Knorpelschäden bzw. noch nicht fortgeschrittener medialer Gonarthrose und tibialem Varus. Ziel ist die Korrektur der mechanischen Beinachse und die Entlastung des medialen Kompartimentes, um die Implantation einer Knietotalendoprothese (Knie-TEP) zu verhindern bzw. weitmöglich hinauszuschieben. Bei richtiger Patientenwahl kann dabei eine deutliche Verbesserung der Schmerzsymptomatik und der Funktion erreicht werden [1, 2]. Dennoch wird bei etwa 30 % der Patienten die Konversion zur Knie-TEP nach HTO notwendig [3]. Idealerweise wird bei der HTO der tibiale metaphysäre Varus korrigiert. Es kann jedoch auch eine Überkorrektur mit unphysiologischer Gelenklinie (medialer proximaler Tibiawinkel [MPTA] über 90°) resultieren [4]. Durch die veränderte Anatomie kann die Operation dann deutlich erschwert sein und zu längeren Operationszeiten durch erschwerte Weichteilbalancierung führen sowie höher gekoppelte Implantate, Stiele und Augmente notwendig machen [5–14].

Primäres Ziel der Untersuchung war der Vergleich der Kniefunktion zwischen Knie-TEP-Patienten mit vorangegangener HTO und bei primärer Gonarthrose 5 Jahre postoperativ. Als weitere Endpunkte wurden das Schmerzniveau, die gesundheitsbezogene Lebensqualität, die Zufriedenheit mit dem Ergebnis der Operation sowie perioperative Daten (Schnitt-Naht-Zeit, Implantattyp, Komplikationen bis 3 Monate postoperativ) und die Überlebenszeit (Revisionen bis 5 Jahre postoperativ) verglichen. Es wurde dabei von der Hypothese ausgegangen, dass Patienten mit vorangegangener HTO weniger von einer Knie-TEP in Bezug auf Funktion, Schmerzlinderung, gesundheitsbezogener Lebensqualität und letztendlich auch Zufriedenheit profitieren.

## Material und Methoden

### Patientenauswahl

**Figure 1:**
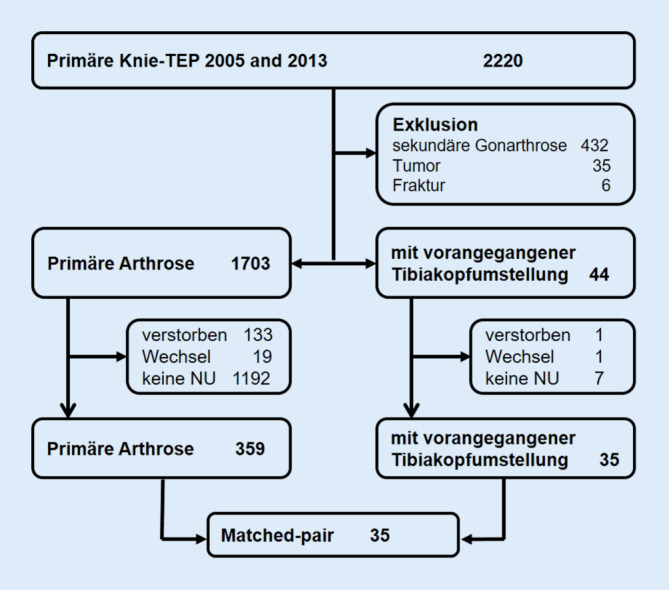

Es erfolgte eine retrospektive Analyse aller Patienten des lokalen Endoprothesenregisters, die zwischen 2005 und 2013 in der Klinik der Autoren einen Kniegelenkersatz erhalten hatten. Eingeschlossen wurden Patienten, die die Knie-TEP nach einer HTO (HTO-Gruppe) oder aufgrund primärer Gonarthrose (PA-Gruppe) erhielten und von denen eine 5‑Jahres-Nachuntersuchung vorlag. Alle Patienten waren prospektiv in das Register eingeschlossen und zur 5‑Jahres-Nachuntersuchung eingeladen worden. Ausgeschlossen wurden Knie-TEP-Operationen aufgrund sekundärer Arthrose (posttraumatisch, inflammatorisch, Osteonekrose), Tumor- und Frakturprothesen sowie Patienten ohne 5‑Jahres-Nachuntersuchung. Insgesamt erfolgten in dem genannten Zeitraum 2220 primäre Knie-TEP. Davon hatten 44 Patienten im Vorfeld eine HTO, entweder in unserer Klinik oder extern. Die HTO war dabei in unterschiedlichen Techniken erfolgt („open-wedge“, „closed-wedge“). Von 35 Patienten (79,5 %) der HTO-Gruppe und von 359 der 1703 Patienten aus der PA-Gruppe (21,1 %) war ein 5‑Jahres-Follow-up verfügbar (Abb. 1).

Um Confounder auszuschließen, wurde eine „propensity score matched-pair“-Analyse durchgeführt. Die Daten von 35 Patienten der HTO-Gruppe wurden mit Daten von 359 Patienten der PA-Gruppe hinsichtlich Geschlecht, Alter, Body-Mass-Index (BMI) und Komorbiditäten (ASA-Score) abgeglichen. 35 Paare konnten identifiziert werden.

### Klinische Untersuchung

Die kniebezogene Funktion wurde prä- und 5 Jahre postoperativ mittels des Knee Society Scores (KSS) [15] erhoben, welcher als separater Function-Score (Gehstrecke, Treppensteigen, Gehhilfen) und Knee-Score (Schmerz, Bewegungsumfang, Stabilität, Fehlstellung) ausgewertet wurde. Es erfolgte zusätzlich die separate Auswertung des im KSS enthaltenen Knieschmerz (Schmerz-Score). Hier wurden 0–50 Punkte in 7 Stufen vergeben, wobei das höchste Schmerzniveau mit einer Punktzahl von 0 bewertet wurde [15].

Prä- und postoperativ erfolgten klinische Untersuchungen mit Messung des Bewegungs-umfanges („range of motion“ [ROM]), der Kniestabilität sowie Röntgenaufnahmen (a.-p., lateral, Ganzbeinaufnahme im Stehen) zur Evaluierung der Beinachse und des MPTA.

Zum 5‑Jahres-Follow-up wurden zusätzlich die gesundheitsbezogene Lebensqualität mit dem SF-36-Health Survey (SF-36) [16, 17] und die allgemeine Zufriedenheit mit dem Ergebnis der Knie-TEP auf einer visuellen Analogskala (VAS) von 0 (völlig unzufrieden) bis 10 (sehr zufrieden) erhoben. Weiterhin erfolgte die Erfassung von Revisionen.

Bei allen Patienten wurden aus den Patientenakten soziodemografische Daten zum Zeitpunkt der Knie-TEP (Alter, Größe, Gewicht, ASA-Score) und perioperative Daten (Schnitt-Naht-Zeit, das verwendete Implantat, Komplikationen in den ersten 3 Monaten postoperativ) ermittelt (Basisdaten).

## Statistische Analyse

Das Matching erfolgte in zwei Schritten, zunächst exaktes Matching, dann „propensity score-matching“. Dabei erfolgte zunächst ein 1:1 Matching für die Variablen Geschlecht und ASA-Score. Im zweiten Schritt wurde für jeden Patienten nach HTO der „nearest neighbour“ ermittelt. Dazu wurde aus den Variablen Alter, BMI, Geschlecht und ASA-Score der Neigungsscore („propensity score“, Wert zwischen 0 und 1) errechnet. Das „propensity score-matching“ erfolgte im Verhältnis 1:1 mit Replacement aus der PA-Gruppe. Die Matched-Pair-Analyse wurde mit der R‑Software, Paket „match-It“ [18, 19] durchgeführt.

Für die Auswertung wurde SPSS Release 24 für Windows (SPSS Inc, Chicago, IL, USA) verwendet. Die Daten wurden als Mittelwert (Standardabweichung) für kontinuierliche Variablen und absolute (relative) Häufigkeiten für kategoriale Variablen dargestellt. Die Vergleiche zwischen den Gruppen basierten auf dem Mann-Whitney-U-Test für kontinuierliche Variablen bzw. auf Chi-Quadrat-Tests für kategoriale Variablen. Die Darstellung der Revisionsraten erfolgte mittels Kaplan-Meier Überlebenskurven, Unterschiede zwischen den Gruppen wurden mit dem Log-Rank Test geprüft. Die Ergebnisse aller Signifikanztests wurden als *p*-Werte zusammengefasst, wobei das akzeptierte Mindestmaß *p* < 0,05 betrug. Aufgrund der Seltenheit und somit limitierten Verfügbarkeit von Knie-TEP nach HTO erfolgte keine Fallzahlplanung, alle verfügbaren Patienten wurden eingeschlossen.

## Ergebnisse

Die Basisdaten beider Gruppen sind in Tab. 1 ersichtlich. Patienten in der HTO-Gruppe im Vergleich zur PA-Gruppe waren zum Zeitpunkt der Knie-TEP signifikant jünger und hatten weniger Komorbiditäten. Die Schnitt-Naht-Zeit nach HTO war signifikant länger und intraoperativ wurde ein signifikant größerer Anteil an modularen Endoprothesen mit Stielverankerung benötigt.

**Table 1:** 

	HTO-Gruppe	PA-Gruppe	*p*-Wert
*n*	44	1703	–
Alter zur Operation (Jahre)	59,8 (SD 11,3)	70,5 (SD 8,8)	**<** **0,001**
Geschlecht weiblich	25 (57 %)	1128 (66 %)	0,193
Body-Mass-Index (kg/m^2^)	31,2 (SD 5,0)	31,1 (SD 5,2)	0,611
*Komorbiditäten*			**0,003**
ASA 1 und 2	32 (73 %)	744 (50 %)	**–**
ASA 3 und 4	12 (27 %)	733 (50 %)	**–**
Schnitt-Naht-Zeit (min)	107 (23)	88 (19)	**<** **0,001**
*Implantattyp*			**<** **0,001**
Ungekoppelt	33 (75 %)	1666 (97,8 %)	**–**
Ungekoppelt mit Stem/Wedge	10 (22,7 %)	3 (0,2 %)	**–**
Gekoppelt	1 (2,3 %)	34 (2,0 %)	**–**

*ASA* American Society of Anesthesiologists

Um Alter, Geschlecht, BMI und ASA-Score als Confounder auszuschließen, erfolgte ein Matched-Pair-basierter Kohortenvergleich.

Die Patienten nach HTO hatten eine signifikant veränderte Anatomie des Tibiakopfes, 50 % hatten einen unphysiologischen MPTA von mehr als 90°. Für die Kniefunktion konnten sowohl prä-, als auch postoperativ keine signifikanten Unterschiede zwischen den gematchten Gruppen gefunden werden. Function Score, Knee Score und ROM zeigten jedoch eine deutliche Verbesserung (Tab. 2). Präoperativ zeigte sich ein signifikant niedrigerer Schmerz-Score und somit ein höheres Schmerzniveau in der HTO-Gruppe im Vergleich zur PA-Gruppe. Die Patienten der HTO-Gruppe verbesserten sich zwar stärker hinsichtlich des Schmerzniveaus, jedoch zeigte sich postoperativ weiterhin ein signifikant höheres Schmerzniveau in der HTO-Gruppe. Bei Betrachtung der gesundheitsbezogenen Lebensqualität zeigten sich in der HTO-Gruppe niedrigere Ergebnisse in den Dimensionen emotionale Rollenfunktion (59,8 vs. 83,8 Punkte; *p* = 0,028) und körperliche Schmerzen (57,6 vs. 73,4 Punkte; *p* = 0,026). Die körperliche Summenskala (39,8 vs. 42,6; *p* = 0,412) und die psychische Summenskala (44,6 vs. 52,9, *p* = 0,098) in deutscher Normierung hingegen zeigten keine signifikanten Unterschiede auf. Beide Gruppen waren 5 Jahre postoperativ mit dem Ergebnis der Knie-TEP sehr zufrieden, die HTO-Gruppe zeigte eine etwas geringere Zufriedenheit.

**Table 2:** 

	HTO-Gruppe gematcht	PA-Gruppe gematcht	*p*-Wert
*n*	35	35	–
Schnitt-Naht-Zeit (min)	107,7 (SD 25,0)	88,1 (SD 20,4)	**0,001**
Abweichung von neutraler Beinachse vor Operation (°)	6,1 (SD 4,7)	8,6 (SD 4,3)	**0,022**
MPTA vor Knie-TEP (°)	90,7 (5,0)	85,6 (2,9)	**<** **0,001**
Anteil MPTA > 90°	50,0 %	5,3 %	**0,002**
*Knee-Score (max. 100 Punkte)*			
Präoperativ	41,9 (SD 14,2)	45,5 (SD 15,0)	0,294
5 Jahre postoperativ	79,2 (SD 17,2)	86,9 (SD 14,3)	0,062
Verbesserung	37,8 (SD 22,7)	41,5 (SD 22,8)	0,530
*Function-Score (max. 100 Punkte)*			
Präoperativ	56,3 (SD 14,8)	53,1 (SD 13,6)	0,359
5 Jahre postoperativ	69,9 (SD 20,0)	71,6 (SD 21,4)	0,886
Verbesserung	13,6 (SD 21,3)	17,4 (SD 20,4)	0,442
*Schmerz-Score (max. 50 Punkte)*			
Präoperativ	10,9 (SD 10,1)	18,7 (SD 11,7)	**0,005**
5 Jahre postoperativ	34,9 (SD 15,3)	40,3 (SD 14,3)	**0,048**
Verbesserung	24,0 (SD 19,7)	21,6 (SD 16,7)	0,536
*ROM*			
Präoperativ	103,3 (SD 17,4)	106,1 (SD 20,3)	0,529
5 Jahre postoperativ	116,9 (SD 14,4)	111,0 (SD 14,3)	0,107
Verbesserung	12,7 (SD 11,8)	5,2 (SD 21,4)	0,091
Zufriedenheit mit dem Operationsergebnis nach 5 Jahren (max. 10 Punkte)	8,0 (SD 1,6)	8,8 (SD 1,4)	0,068

*HTO* Hohe Tibiakopfumstellungsosteotomie, *MPTA* medialer proximaler Tibiawinkel, *PA* Patienten mit primärer Gonarthrose, *ROM* „range of motion“, *TEP* Totalendoprothese

Hinsichtlich der postoperativen Komplikationen innerhalb der ersten 3 Monate nach Operation unterschieden sich die gematchten Gruppen nicht signifikant. Drei Patienten (8,5%) in der HTO-Gruppe und vier Patienten (11,4 %) der PA-Gruppe entwickelten postoperativ eine 1‑Etagen-Beinvenenthrombose des ipsilateralen Unterschenkels (*p* = 0,690).

Die kumulative revisionsfreie 5‑Jahres-Überlebensrate der Knie-TEP lag bei Patienten nach HTO bei 95,3 % und bei Patienten mit primärer Gonarthrose bei 98,5 %. Der Unterschied war nicht signifikant (*p* = 0,448, Log Rank Test). Innerhalb von 5 Jahren wurde bei 3 Patienten nach HTO ein Revisionseingriff durchgeführt. Es erfolgten zwei vollständige Wechseloperationen sowie ein Femurteilwechsel aufgrund ausgeprägter Ossifikationen mit konsekutivem Beuge- und Streckdefizit.

## Diskussion

Als Hauptergebnis der Studie zeigte sich, dass Patienten nach Knie-TEP mit vorangegangener HTO eine gleichwertige Kniefunktion, gesundheitsbezogene Lebensqualität und Zufriedenheit im mittelfristigen Follow-up erzielen konnten wie Patienten in einer gematchten Kontrollgruppe mit Knie-TEP aufgrund primärer Gonarthrose. Jedoch zeigte die HTO-Gruppe signifikant schlechtere Schmerzscore-Werte (prä- und postoperativ). Hinsichtlich des intraoperativen Mehraufwandes konnte diese Studie bereits veröffentlichte Arbeiten bestätigen. Dieser Mehraufwand zeigte sich in der durchschnittlich 20 min längeren Operationszeit und deutlich häufiger notwendigen modularen Endoprothesen mit Stielverankerung. Dabei sind die Gelenkdarstellung bei postoperativer Narbenbildung, die Weichteilbalancierung bei unphysiologischer Anatomie des Tibiakopfes und die Ausrichtung und Verankerung der tibialen Komponente als zentrale technische Herausforderungen während der Operation beschrieben [8, 20–23].

Konsistent zu den Daten großer Registeruntersuchungen und systematischen Reviews [5, 12, 22, 24] zeigte sich die HTO-Gruppe mit einem signifikant größeren Anteil an jungen, männlichen und gesunden Patienten. Es ist anzunehmen, dass in der Literatur beschriebene Unterschiede in den Ergebnissen nach Knie-TEP durch diese Gruppenunterschiede verzerrt werden. Daher sind die Ergebnisse von Studien, die ein quasi randomisiertes Design via Matching durchführen, deutlich aussagekräftiger.

Entsprechend den Ergebnissen von El-Galaly et al. [7], welche Unterschiede der Ergebnisse bei Knieendoprothesen bei primärer Gonarthrose versus denen nach HTO auf die Alters- und Geschlechterverteilung zurückführten, erfolgte der Ausschluss dieser Confounder durch Matching zweier vergleichbarer Kohorten.

Nach Matching zeigte sich konsistent zu anderen Studien [9, 10, 12, 22], dass Patienten nach HTO eine vergleichbare postoperative Kniefunktion (ROM, Knee-Score und Function-Score) erreichten wie Patienten der PA-Gruppe. Allerdings war das Schmerzniveau bei Patienten mit vorangegangener HTO nach der Knie-TEP höher, was ebenfalls in anderen Arbeiten [25, 26] und in einer kürzlich veröffentlichten Metaanalyse [27] gezeigt werden konnte. Eine denkbare Erklärung könnte die gezeigte Häufung einer Patella baja bei Patienten nach HTO (insbesondere bei „Open-wedge“-Technik) mit konsekutivem vorderem Knieschmerz liefern [11]. Weitere mögliche Erklärung sind extensives Weichteilrelease, Malalignment der Patella sowie ein Schaftspitzenschmerz der deutlich häufiger vorhandenen Stielverankerung [25, 27]. Das bereits vor der Knie-TEP höhere Schmerzniveau bei Patienten nach HTO und die in beiden Gruppen in etwa gleichem Ausmaß erzielte individuelle postoperative Schmerzreduktion durch die Knie-TEP spricht jedoch gegen Ursachen im Rahmen der Knie-TEP Implantation. Insofern könnten präoperative Vernarbungen, Patella baja und möglicherweise die längere Schmerzdauer (Schmerzgedächtnis) hier die wesentlichen Faktoren für das höhere prä- und postoperative Schmerzniveau sein. Trotz des höheren Schmerzniveaus profitierten Patienten nach HTO signifikant von der Knie-TEP, zeigen in dieser Studie jedoch eine etwas geringere Zufriedenheit. Die Zufriedenheit mit der Operation hängt wesentlich von der Erwartungserfüllung der Patienten ab [28–30]. Die Auseinandersetzung mit den Erwartungen von Patienten vor einer Knie-TEP ist somit ein entscheidender Einflussfaktor. In einer kürzlich publizierten Studie konnte gezeigt werden, dass eine strukturierte Auseinandersetzung und Modifikation von Erwartungen zu einer höheren Zufriedenheit führt [31]. Deshalb sollten Operateure das zu erwartende höhere Schmerzniveau mit den Patienten im Rahmen der partizipativen Entscheidungsfindung zur Knie-TEP besprechen.

Hinsichtlich der Revisionsrate und somit der Prothesenstandzeiten gibt es in der Literatur sehr widersprüchliche Ergebnisse. Einerseits fanden einige Registerstudien und Metaanalysen [7, 22, 32] keine signifikant erhöhten Revisionsraten bei Patienten nach Umstellungsosteotomie. Andererseits zeigten andere Arbeiten [5, 12, 33, 34] und eine kürzlich veröffentlichte umfassende Metaanalyse [27] signifikant erhöhte Komplikations- und Revisionsraten. Eine Übersicht über die Revisionsraten einzelner Publikationen gibt Tab. 3. Diese werden in den Arbeiten mit den oben genannten technischen Herausforderungen erklärt, welche den Operateur teilweise zu intraoperativen Kompromissen zwingen. In der untersuchten Kohorte eines spezialisierten Endoprothetikzentrums fanden sich bei limitierter Fallzahl keine erhöhten Komplikationen oder Revisionen innerhalb von 5 Jahren. Dies spricht dafür, diese anspruchsvollen Operationen in spezialisierten Zentren durchzuführen.

**Table 3:** 

Studie	Jahr	Studientyp	Gesamtzahl	Revisionen	Revisions-rate (in %)	Durchschnittliches Follow-up (in Jahren)
Haddad [9]	2000	Retrospektiv	50	6	12,0	6,2
Karabatsos [10]	2002	Retrospektiv	22	0	0,0	5,2
Haslam [35]	2007	Retrospektiv	51	11	21,6	> 5
Kazakos [11]	2008	Retrospektiv	38	0	0,0	4,5
Amendola [25]	2010	Prospektiv	29	4	13,8	8
Efe [6]	2010	Retrospektiv	41	4	9,8	7
Erak [8]	2011	Retrospektiv	34	0	0,0	3,4
Meding [36]	2011	Retrospektiv	39	6	15,4	14
Pearse [12]	2012	Retrospektiv	711	45	6,3	Unbekannt
Niinimaki [33]	2014	Register	1036	93	9,0	> 1
Badawy [5]	2015	Register	1399	83	5,9	Unbekannt
Robertsson [34]	2016	Register	119	3	2,5	> 3
El-Galaly [7]	2018	Register	1044	98	9,4	Unbekannt
Vorliegende Arbeit	2020	Prospektiv	44	2	4,5	5

Limitierungen dieser Arbeit liegen im Studiendesign und der relativ kleinen Fallzahl. Aufgrund der insgesamt geringen Anzahl von Knie-TEP nach HTO war die retrospektive Auswertung von prospektiv erhobenen Daten notwendig. Um diesbezüglich jedoch Confounder bestmöglich auszuschließen, erfolgte die Auswertung als Matched-Pair-Analyse. Weiterhin besteht die Gefahr eines Selektionsbias, einerseits durch die Zuweisung an eine spezialisierte Einrichtung, andererseits dadurch, dass nicht alle Patienten nachuntersucht werden konnten. Auch ist nicht auszuschließen, dass die Erfahrung des Operateurs einen Einfluss auf die Ergebnisse der Knie-TEP nach HTO hatte, da diese Operation üblicherweise durch erfahrene Operateure erfolgt. Für eine abschließende Bewertung der Revisionsraten ist der Nachbeobachtungszeitraum von 5 Jahren sicher nicht ausreichend. Allerdings erfolgen die häufigsten Revisionen aufgrund operationstechnischer Schwierigkeiten innerhalb der ersten 3 Jahre, sodass dies in der vorliegenden Arbeit enthalten ist.

Zusammengefasst können Patienten nach HTO im gleichen Ausmaß von einer Knie-TEP profitieren wie Patienten mit primärer Gonarthrose. Die Operation ist allerdings anspruchsvoller. Hierauf sollte der Operateur vorbereitet sein und auch Implantate mit Stielverlängerung und höherem Kopplungsgrad in Bereitschaft halten. Die Patienten sollten im Vorfeld über das zu erwartende höhere Schmerzniveau informiert werden.

## Fazit für die Praxis

- Patienten nach Tibiakopfumstellungsosteotomie (HTO) können im gleichen Ausmaß von einer Knietotalendoprothese (Knie-TEP) profitieren wie Patienten mit primärer Gonarthrose.
- Aufgrund des höheren prä- und postoperativen Schmerzniveaus sollte der Operateur mit den Patienten die Erwartungen an den Eingriff präoperativ genau besprechen.
- Die Implantation einer Knie-TEP nach HTO ist deutlich anspruchsvoller.
- Modulare Implantate mit der Möglichkeit von Stielverlängerung, Knochendefektaugmentation und höherem Kopplungsgrad sollten vorrätig sein.

## Abkürzungen

ASA: American Society of Anesthesiologists
BMI: Body-Mass-Index
HTO: Hohe Tibiakopfumstellungsosteotomie
KSS: Knee Society Score
MPTA: Medialer proximaler Tibiawinkel
PA: Patienten mit primärer Gonarthrose
ROM: „Range of motion“
SD: Standardabweichung
SF: Short Form
TEP: Totalendoprothese
VAS: Visuelle Analogskala
